# *Lactobacillus paracasei* L9 Ameliorates Pulmonary Fibrosis in Aged Mice via Gut‐Lung Axis‐Mediated Regulation of Immune Cell Migration

**DOI:** 10.1111/acel.70576

**Published:** 2026-06-08

**Authors:** Ran Bi, Yiran Zhang, Wen Zhang, Chenhong Shi, Ziyu Qiao, Rui Quan, Yanan Sun, Juan Chen, Ran Wang, Fazheng Ren, Yixuan Li

**Affiliations:** ^1^ Key Laboratory of Precision Nutrition and Food Quality, Department of Nutrition and Health China Agricultural University Beijing China; ^2^ Food Laboratory of Zhongyuan Luohe China

**Keywords:** collagen I, gut‐lung axies, *Lactobacillus paracasei* L9, pulmonary fibrosis, Th17 cell

## Abstract

Age‐related pulmonary fibrosis (PF) imposes a growing global burden with limited therapies. This study explored the role of *Lactobacillus paracasei* L9 (L9) in alleviating PF in C57BL/6J mice and its mechanisms. Nine‐month oral administration of L9 (4 × 10^9^ CFU/mL) suppressed collagen (Col‐) I deposition in aged mice, with no significant effect on Col‐III. Mechanistically, L9 inhibited the JNK‐HSF1 signaling pathway, thereby resulting in a 61% decrease in HSP47 expression, which is crucial for Col‐I synthesis. L9 reshaped the gut microbiota by increasing short‐chain fatty acid (SCFA)‐producing bacteria (e.g., Blautia), leading to a 97% increase in serum propionic acid and 193% increase in butyric acid; notably, the levels of SCFAs in the lungs were below the limit of detection. In L9‐treated mice, pulmonary IL‐17A levels and Th17 cell populations were reduced. In vitro, SCFAs directly inhibited Th17 cell differentiation and IL‐17A secretion, and IL‐17A was confirmed to promote Col‐I synthesis via the JNK‐HSF1 pathway in pulmonary fibroblasts. Consequently, L9 modulates the gut microbiota to produce SCFAs, which regulate naïve CD4^+^ T cell differentiation and migration via the gut‐lung axis. This reduces pulmonary Th17 cells and IL‐17A, thereby suppressing Col‐I synthesis in pulmonary fibroblasts and ultimately alleviating age‐related PF. In conclusion, this study highlights L9 as a microbiome‐targeted precision nutrition strategy for the adjuvant therapy of age‐related PF via the novel gut‐lung axis mechanism.

## Graphical abstract

Oral administration of *Lactobacillus paracasei* L9 (L9) reshapes the gut microbiota, specifically increasing the abundance of short‐chain fatty acid (SCFA)‐producing bacteria (e.g., Blautia), thereby leading to a significant elevation of serum propionic acid (97% increase) and butyric acid (193% increase). Through the gut‐lung axis—a novel regulatory pathway identified in this study—SCFAs modulate the differentiation of naive CD4^+^ T cells, consequently reducing the population of pulmonary Th17 cells and the secretion of IL‐17A. Notably, IL‐17A was confirmed to promote collagen type I (Col‐I) synthesis via the JNK‐HSF1 signaling pathway in pulmonary fibroblasts; thus, the reduction of IL‐17A inhibits this pro‐fibrotic pathway, resulting in a 61% decrease in HSP47 expression (a key regulator of Col‐I synthesis) and ultimately suppressing Col‐I deposition in the lungs. This cascade collectively mitigates age‐related pulmonary fibrosis, highlighting L9 as amicrobiome‐targeted precision nutrition strategy for the adjuvant therapy of pulmonary fibrosis via the novel gut‐lung axis mechanism (created in https://BioRender.com).

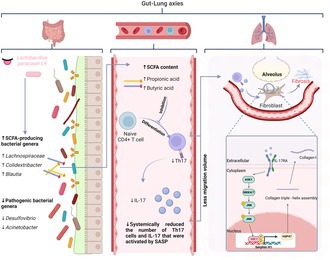

## 1 Introduction

In recent years, numerous studies have shown the lung may develop fibrosis with age (Moss et al. 2022). And because of the aggravation of global aging, the medical burden of pulmonary fibrosis (PF) increases rapidly (Hutchinson et al. 2015). Most patients with PF have a slow progressive decline in lung function that eventually leads to respiratory failure, while 10%–15% of patients experience an unusually rapid decline over several months (Ley et al. 2011). In the past few years, the main pathogenesis of PF has been clearly elucidated: myofibroblasts are abnormally activated and over‐secrete extracellular matrix (ECM) after repeated injury of alveolar epithelium, which eventually leads to lung structural remodeling (Henderson et al. 2020). Collagen is the primary constituent of the ECM in the lung, which contributes to normal wound healing (Tomasek et al. 2002). However, the ability of the aging lung to inactivate myofibroblasts and balance collagen synthesis and degradation is significantly weakened; that is why the aging lung is more susceptible to fibrosis (Moss et al. 2022).

Therefore, maintaining the balance of collagen synthesis and degradation plays a key role in the prevention of PF (Henderson et al. 2020). The synthesis of collagen proteins entails the production and release of collagen precursor forms, along with the assembly of collagen fibers (Hulmes 2002). Many different enzymes are involved in the production of collagen (Shoulders and Raines 2009). The molecular chaperone heat shock protein 47 (HSP47), for instance, plays a vital role in facilitating the correct folding of procollagen molecules during the synthesis of collagen I (Col‐I) (Sakamoto et al. 2023). During the assembly of mature collagen fibers, co‐binding occurs between or with other molecules to improve the stability and mechanical strength of the collagen fiber (Hulmes 2002). In the degradation of collagen, the matrix metalloproteinase (MMP) family of enzymes and cathepsin K are the main enzymes (Sprangers and Everts 2019). Tissue anomalies and collagen dyshomeostasis are the results of these enzyme or molecular chaperone abnormalities. Hence, an improved comprehension of the alterations in these enzymes and molecular chaperones in the aging lung may provide new targets for therapeutic intervention aimed at mitigating PF.

Currently, the only pharmacotherapies available for the treatment of PF are the costly drugs nintedanib and pirfenidone, which may produce adverse effects such as liver function impairment and headaches. The dietary intervention offers the advantages of better nutrition support and fewer side effects in comparison. It has been reported that the implementation of nutritional intervention strategies for elderly patients to affect the diversity of intestinal flora and its metabolites is a potential adjuvant therapy (Dang and Marsland 2019). Furthermore, research indicated that alterations in gut microbiota and associated by‐products, such as endotoxin, metabolites, cytokines, and hormones, may enter the lungs through the bloodstream and impact inflammation and immune responses related to lung diseases (Zhang et al. 2020). Therefore, concerns have recently arisen regarding the potential that nutritional intervention to regulate intestinal flora and its by‐products is a potentially effective way to alleviate PF (Chioma et al. 2022).

*Lactobacillus*, as a natural biological agent, has a beneficial function of regulating respiratory tract defenses (Strauss et al. 2021). Oral *Lactobacillus* has been shown in numerous trials to ameliorate respiratory conditions, such as cystic fibrosis, asthma, cancer, and chronic obstructive pulmonary disease (Du et al. 2022). *Lactobacillus paracasei* L9 (L9) (CGMCC No. 9800), which was first isolated from the feces of healthy centenarians, has been demonstrated to reduce the symptoms of allergies in mice models (Yang et al. 2015). Wang et al. (2017) showed that oral administration of L9 may reduce airway hyperresponsiveness and allergic airway response in asthmatic mouse models by rebalancing Th1/Th2 immune response and regulating IL‐17 pro‐inflammatory immune response. However, there is no research exploring if L9 can alleviate PF currently. Thus, this study aims to determine whether L9 can mitigate the effects of aging‐related PF and to further clarify the important role of intestinal flora and its metabolites in the disease.

## 2 Results (or Results and Discussion)

### 2.1 PF Markers Are Increased in the Lung Tissue of Aging Individuals

**FIGURE 1:**
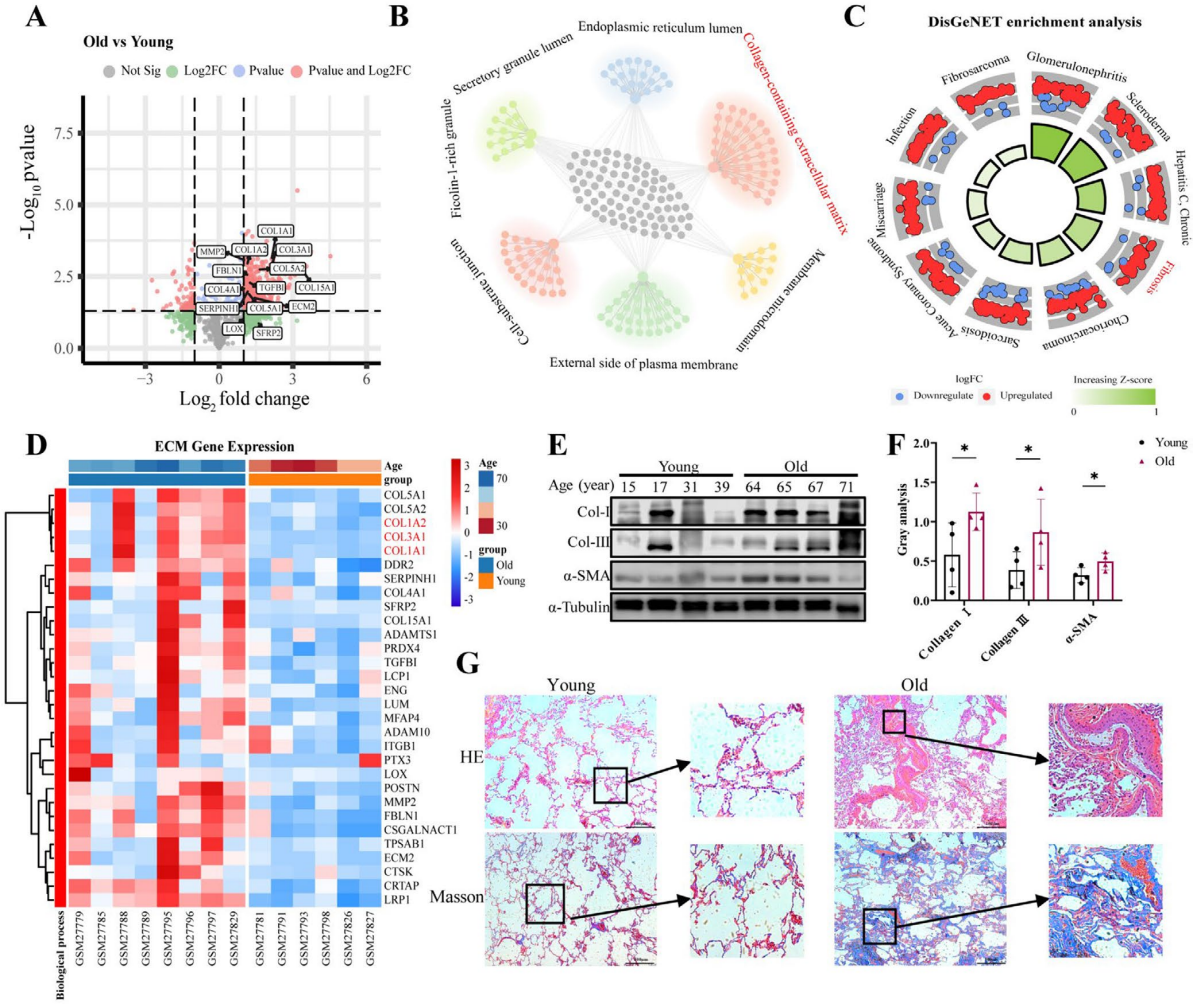
Increased PF markers in the lung tissue of elderly individuals. (A) Volcano plot of the DEGs of young and old donors. (B) Gene Ontology (GO) enrichment analysis. (C) Gene‐disease association enrichment analysis. (D) Heatmap of differential expression analysis of key ECM deposition‐related proteins in the lungs of young and old groups. (E) The expression levels of Col‐I, Col‐III, α‐SMA, and α‐Tubulin were detected using Western blot analysis. (F) α‐Tubulin was used as the internal reference for Col‐I, Col‐III, and α‐SMA. Relative protein expression levels were determined using gray scale analysis. Three independent experiments were performed, *n* = 4. Data was expressed as mean ± SD. Statistical analysis was conducted using independent samples *t*‐test. (G) HE and Masson staining of lung tissue, magnification, 50×. Scale bar = 200 μm. * *p* < 0.05.

Our previous research indicated that aging is a major catalyst in the development of PF, with PF primarily occurring in individuals aged 50 and above (Quan et al. 2024). However, most studies group subjects based on disease status, overlooking the fact that PF is the outcome of long‐term, complex interactions and carries a high risk of delayed diagnosis. To further clarify the isolated impact of age on the PF, this study focuses on the lung tissue of young and aging individuals. We analyzed publicly available bulk RNA sequencing data from the NCBI GEO platform (accession number GSE1643), which includes 17 normal lung tissue donors, comprising 9 young donors and 8 older donors. Based on |Log_2_ (Fold Change)| > 1, 1217 differentially expressed genes (DEGs) were identified, with 468 DEGs significantly upregulated and 86 DEGs significantly downregulated in the old group (Figure 1A). Combined with the GO analysis results, there was a significant upregulation in the expression of genes related to ECM structural constituent (Figure 1B). Additionally, the results of the Gene‐disease association (DisGeNET) enrichment analysis indicated that the upregulated genes in the lung were indicative of a high risk of fibrosis development (Figure 1C). Furthermore, we created a clustered heatmap of gene expression for ECM components and found that aging leads to significant expression of ECM genes (Figure 1D). To validate these findings, we examined lung tissues from eight donors. Initially, we assessed Col‐I and Col‐III (the main proteins deposited in ECM) and α‐SMA (PF protein markers) in lung tissues. The results indicated that levels of Col‐I, Col‐III, and α‐SMA were significantly higher in the old group compared to the young group (*p* < 0.05) (Figure 1E,F). Finally, we conducted pathological and Masson's trichrome staining observations. The results revealed significant airway remodeling in the old group, with thickened pulmonary parenchyma and extensive fibrotic areas. Blue‐stained collagen fibers replaced normal alveolar structures, and some regions exhibited a honeycomb‐like appearance (Figure 1G).

### 2.2 PF Markers Are Increased in the Lung Tissue of Aging Mice, With Collagen Deposition Being the Main Contributing Factor

**FIGURE 2:**
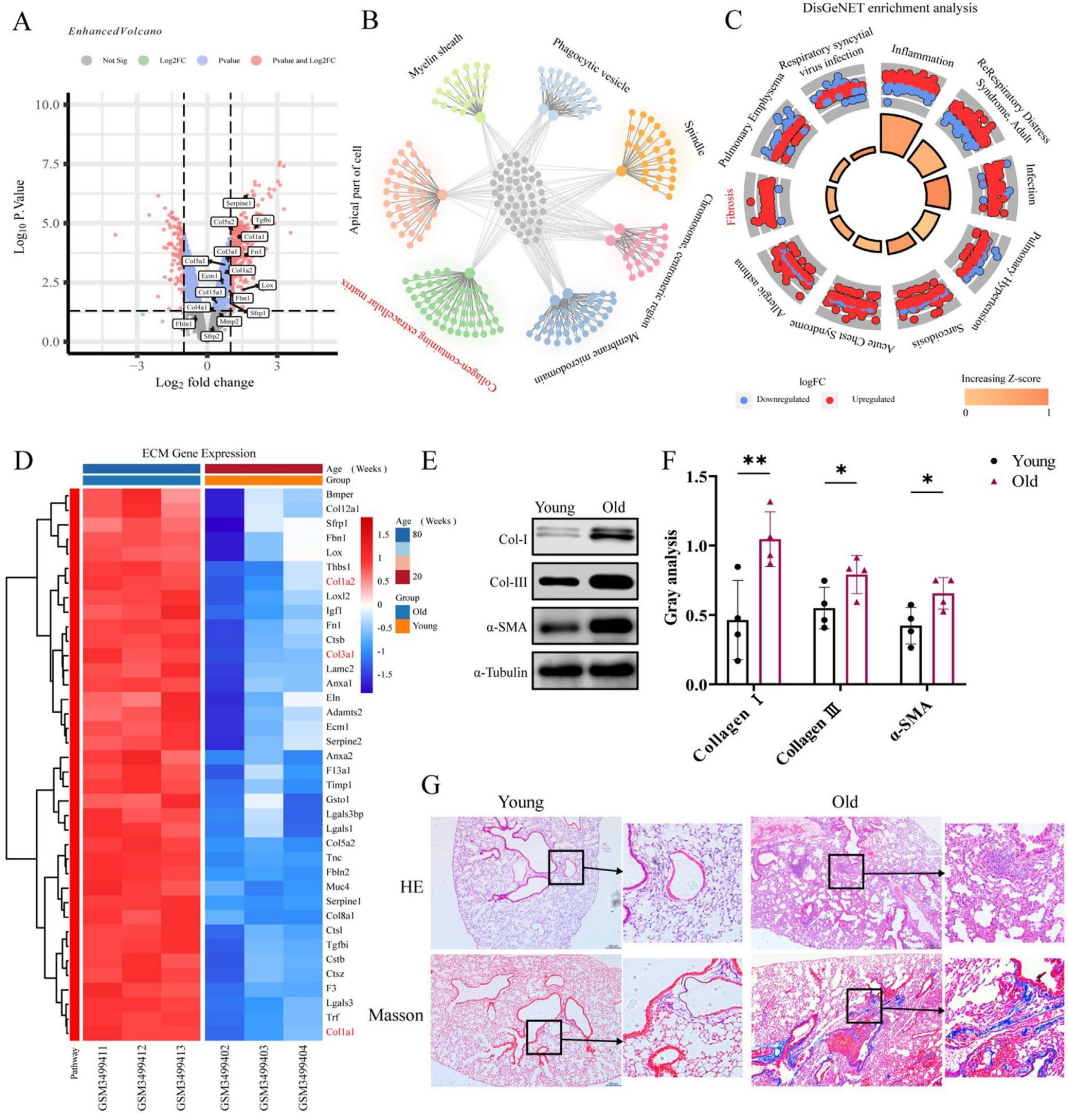
Increased PF markers in the lung tissue of aging mice. (A) Volcano plot of the DEGs of young and old mice. (B) GO enrichment analysis. (C) Gene‐disease association enrichment analysis. (D) Heatmap of differential expression analysis of key ECM deposition‐related proteins in the lungs of young and old groups. (E) The expression levels of Col‐I, Col‐III, α‐SMA, and α‐Tubulin were detected using Western blot analysis (*n* = 4). (F) α‐Tubulin was used as the internal reference for Col‐I, Col‐III, and α‐SMA. Relative protein expression levels were determined using gray scale analysis, *n* = 4. Data was expressed as mean ± SD. Statistical analysis was conducted using independent samples *t*‐test. (G) HE and Masson staining of lung tissue, magnification, 50×, *n* = 6. Scale bar = 200 μm. * *p* < 0.05, ** *p* < 0.01.

After establishing that the expression of PF markers in human lung tissues significantly increases during aging, we conducted a comprehensive and thorough study using a mouse model. Initially, we utilized public databases from the NCBI GEO platform (accession number GSE123293), which include lung tissues from 3 mice aged 16 weeks and 3 mice aged 82 weeks. Analysis of DEGs identified 360 significantly upregulated and 127 significantly downregulated genes in aged mouse lung tissue (Figure 2A). Consistent with findings in human populations, GO enrichment analysis demonstrated significant upregulation of ECM genes (Figure 2B). Meanwhile, the results of DisGeNET enrichment analysis indicated that the upregulated genes in mouse lungs also suggest an increased risk of fibrosis (Figure 2C). The expression of Col‐I and Col‐III was significantly increased in old mice (*p <* 0.05) (Figure 2D). Western blot analysis demonstrated significantly elevated protein expression levels of Col‐I, Col‐III, and α‐SMA in the aged group (*p <* 0.05) (Figure 2E,F). Further pathological analysis revealed increased lung density, alveolar structural damage, and significant collagen deposition in 24 M mice, indicative of a PF phenotype (Figure 2G). Therefore, the increased collagen deposition is the main contributing factor for PF in aging mice.

### 2.3 L9 Alleviates Fibrosis Through Col‐I Deposition in Aging Mice, Rather Than Col‐III

Recently, some researchers linked *Lactobacillus* to the synthesis of collagen (Yu et al. 2024). According to previous research, L9, a probiotic derived from the intestines of long‐lived, healthy old adults, enhances lung health (Wang et al. 2017). Based on the aforementioned results, aging individuals have a higher propensity to develop PF, which is primarily characterized by collagen deposition. Therefore, it is crucial to clarify whether L9 plays a significant role in collagen deposition during PF under the natural aging model.

**FIGURE 3:**
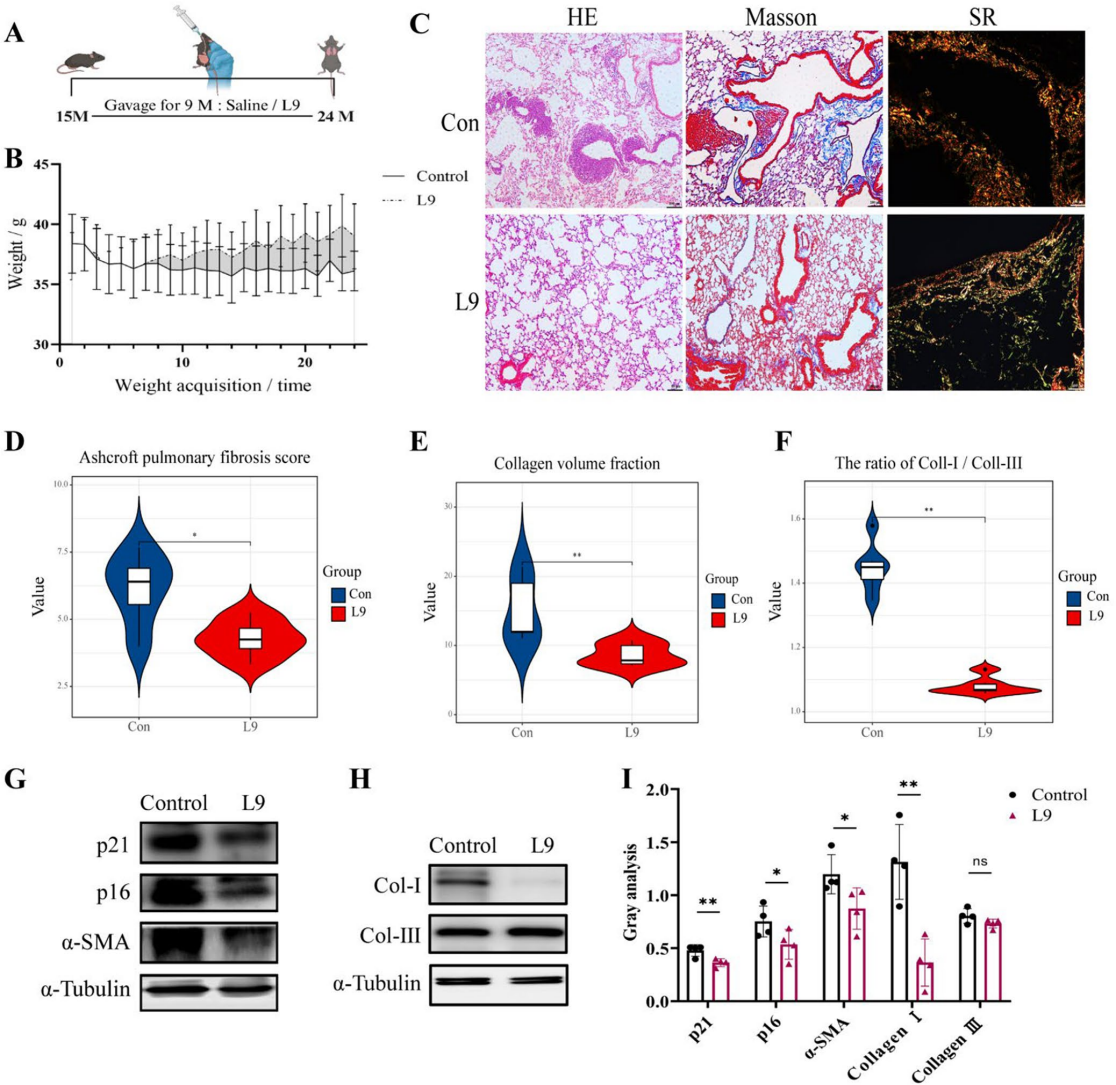
Collagen deposition and fibrosis are reduced in aged mice by L9. (A) Operation flowchart and research design; *n* = 8 animals per group. (B) Body weight of mice. (C) H&E staining, Masson staining, and Sirus red staining of lung tissue, magnification, 100×. (D) Ashcroft PF score based on H&E staining, *n* = 6. Scale bar = 100 μm. (E) Collagen volume fraction based on Masson staining, *n* = 6. Scale bar = 100 μm. (F) Polarizing microscope view the ratio of red light and green light. Scale bar = 100 μm. (G, H) The expression levels of p21, p16, α‐SMA, Col‐I, Col‐III, and α‐Tubulin were detected using Western blot analysis. (I) α‐Tubulin was used as the internal reference for p21, p16, α‐SMA, Col‐I, and Col‐III. Relative protein expression levels were determined using gray scale analysis. Three independent experiments were performed. Data was expressed as mean ± SD; *n* = 4. Statistical analysis was conducted using independent samples *t*‐test. * *p* < 0.05, ** *p* < 0.01.

To ascertain if L9 influences collagen‐related PF symptoms, we initially conducted a comprehensive investigation utilizing a 15 to 24 M natural aging mouse model (Figure 3A). Weekly body weight measurements were taken during the research to assess the general health of the mice. It was found that the weight of aged mice remained stable after L9 intervention (Figure 3B). Lung histopathology was evaluated to determine whether the L9 intervention alleviated lung structure damage. The lung alveolar structure was transparent and there was no collagen protein accumulation of 15 M mice (Figure 3C). Nevertheless, the lungs of 24 M mice showed significant accumulation of collagen fibers, indicating a developmental path from the periphery to the central lung region, along with severe inflammation and alveolar destruction. Meanwhile, additional collagen fibers were deposited in the damaged areas of trachea and alveoli. In contrast, 24 M mice fed with L9 had reduced inflammatory damage to their lungs (Figure 3C). L9 intervention significantly decreased the lung tissue fibrosis score by 30% (*p <* 0.05) according to the Ashcroft score (Figure 3D). Strikingly, mice in L9 group displayed an enormous reduction in collagen fiber deposition by 40% (*p <* 0.05) (Figure 3E). Sirius Red staining (Figure 3C) demonstrated that the Col‐I to Col‐III ratio dramatically decreased following L9 treatment (*p <* 0.05). Furthermore, we examined the amount of the Col‐I and Col‐III collagen protein (Figure 3H,I). While Col‐III remained largely unaltered, the total amount of Col‐I in the L9 group dropped by 59% compared to the control group (*p <* 0.05). These findings indicate that Col‐I is the primary target through which L9 exerts its effects. Notably, we discovered that L9 significantly lower α‐SMA protein expression (*p <* 0.05), a marker of PF remission (Figure 3F). Concurrently, L9 significantly reduced the expression of senescence markers p21 and p16 (*p <* 0.05) (Figure 3F,I).

### 2.4 L9 Reduces the Amount of Col‐I via Inhibiting Its Synthesis

**FIGURE 4:**
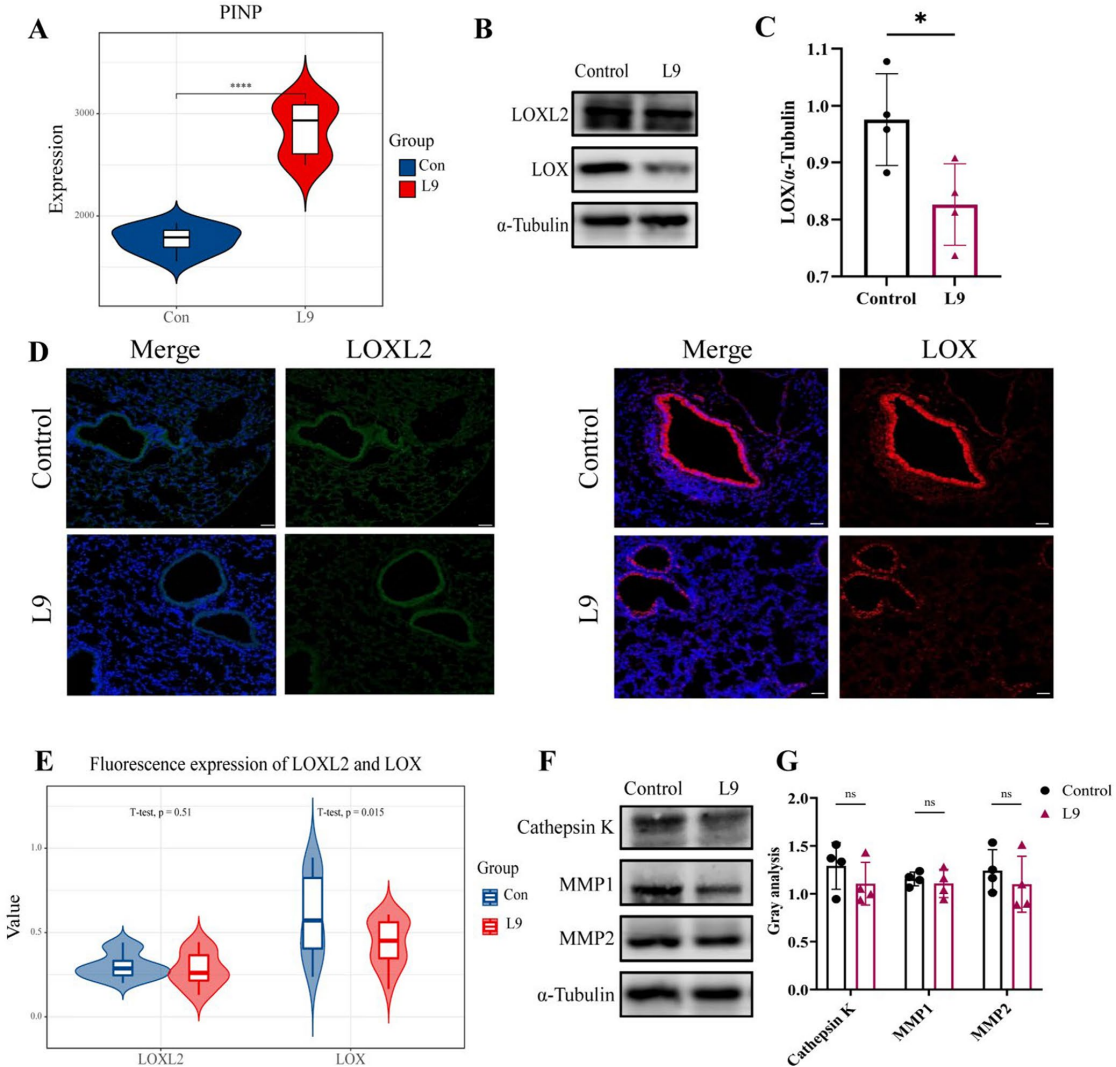
L9 lowers the amount of Col‐I by preventing the synthesis process. (A) The expression levels of PINP in lung tissue. Three independent experiments were performed, *n* = 6. (B, F) The expression levels of LOXL2, LOX, Cathepsin K, MMP1, MMP2 and α‐Tubulin were detected using Western blot analysis. (C, G) α‐Tubulin was used as the internal reference for LOXL2, LOX, Cathepsin K, MMP1 and MMP2. Relative protein expression levels were determined using gray scale analysis. Three independent experiments were performed, *n* = 4. (D) Lung sections were stained with immunofluorescence for LOXL2 and LOX proteins, magnification, 100×. (E) The fluorescence intensity of LOXL2 and LOX protein was analyzed by immunofluorescence staining, *n* = 6. Statistical analysis was conducted using independent samples *t*‐test. Scale bar = 50 μm. (F) The expression levels of Cathepsin K, MMP1, MMP2, and α‐Tubulin were detected using Western blot analysis.* *p* < 0.05, **** *p* < 0.0001.

To establish how L9 influences the amount of Col‐I in the lungs of mice, we explored proteins involved in collagen synthesis, crosslinking, and degradation. Firstly, we found that the overall level of PINP, one of the key ingredients in the Col‐I synthesis process, was drastically lowered by 61% in the L9 group (*p <* 0.05) (Figure 4A). Members of the ECM cross‐linking enzyme family, LOXL2 and LOX, have long been thought to be viable targets for anti‐fibrosis treatments (Chen et al. 2019). Next, we examined them (Figure 4B,C) and found that LOXL2 had no discernible effect whereas L9 dramatically reduced LOX expression levels by 27% (primarily around the trachea) (*p <* 0.05). Furthermore, immunofluorescence was performed to further verify the effect of L9 on LOX (Figure 4D). It was found that L9 significantly reduced LOX expression around the airways by 37% (Figure 4E), which was consistent with the Western blot results. Finally, we investigated the key degradation enzymes of Col‐I, and the results (Figure 4F,G) showed that after the treatment with L9, the expression of Cathepsin K, MMP1, and MMP2 showed a lowering tendency, but with no statistically significant differences. Overall, L9 had a greater influence on Col‐I synthesis.

### 2.5 L9 Inhibits the Expression of Molecular Chaperone HSP47 and Thus Suppresses Its Synthesis

**FIGURE 5:**
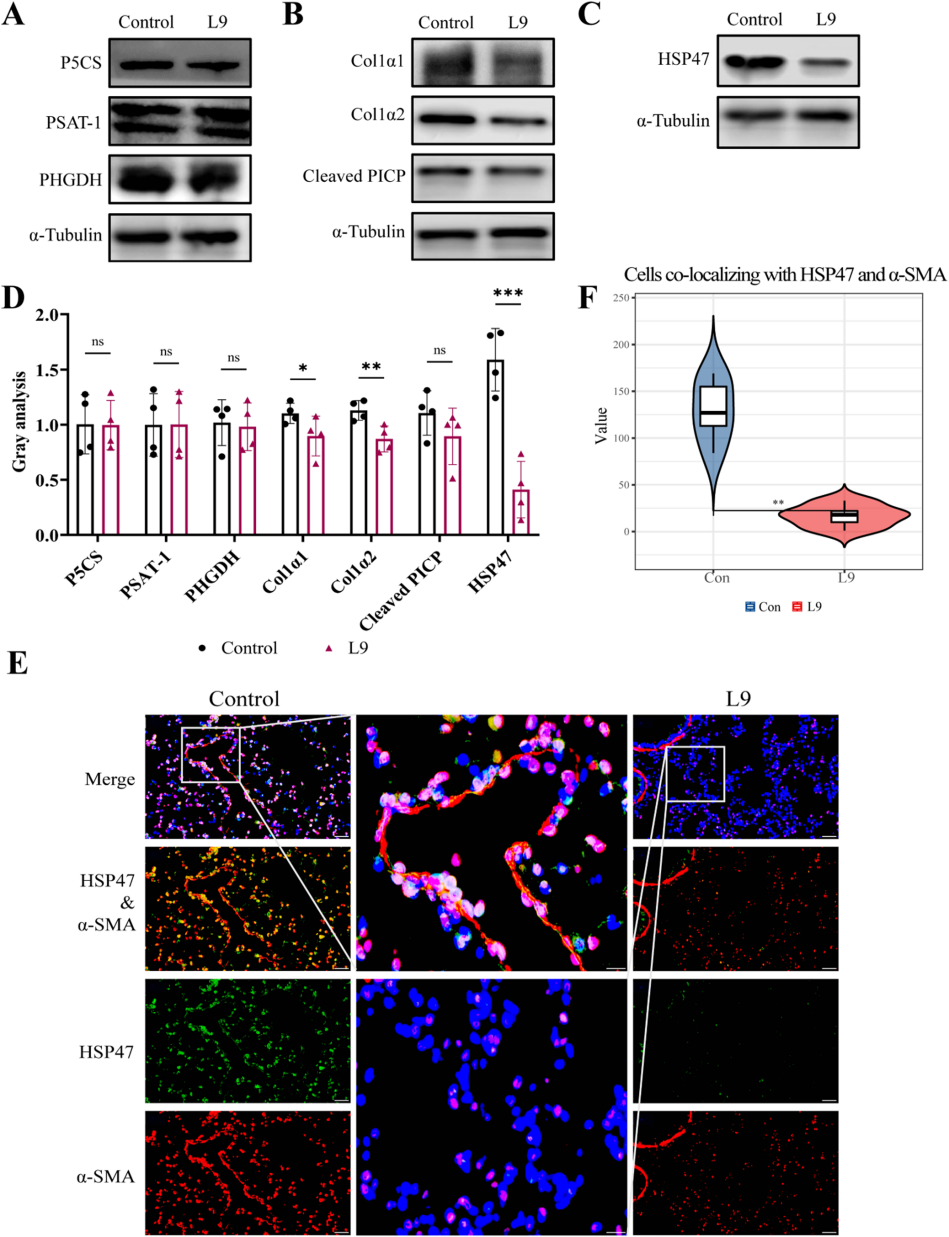
The expression of the molecular chaperone HSP47 is inhibited by L9. (A–C) The expression levels of P5CS, PSAT‐1, PHGDH, Col1α1, Col1α2, cleaved PICP, HSP 47, and α‐Tubulin were detected using Western blot analysis. (D) α‐Tubulin was used as the internal reference for P5CS, PSAT‐1, PHGDH, Col1α1, Col1α2, cleaved PICP and HSP47. Relative protein expression levels were determined using gray scale analysis. Three independent experiments were performed, *n* = 4. (E) Lung sections were stained with immunofluorescence for HSP47 and α‐SMA, magnification, 100×. *n* = 4. Statistical analysis was conducted using independent samples *t*‐test. Scale bar = 50 μm. (F) Counting of cells co‐localizing with HSP47 and α‐SMA, *n* = 5. Statistical analysis was conducted using independent samples *t*‐test. Scale bar = 50 μm. * *p* < 0.05, ** *p* < 0.01, *** *p* < 0.001.

To highlight how L9 impacts Col‐I production, this study examined different nodes of precursor collagen synthesis prior to PINP cleavage. Firstly, we determined the main enzymes that affect the synthesis of serine and proline. The results (Figure 5A,D) demonstrated that L9 did not significantly suppress the protein expression levels of P5CS, PSAT‐1, and PHGDH. Subsequently, we identified the propeptide of C‐Propeptide, Col1α1 chain, and Col1α2 chain of pro‐collagen molecules. The outcomes demonstrated that while L9 intervention led to a declining pattern in the protein expression levels, it did not have significance (Figure 5B,D). Subsequently, we focused on the molecular chaperone HSP47, which is implicated in the folding of collagen in the endoplasmic reticulum. The findings (Figure 5C) demonstrated that L9 dramatically suppressed 61% of HSP47 expression (*p <* 0.05). Hence, L9 primarily controls collagen synthesis through influencing HSP47 expression. To further verify that L9 indeed affects the expression of the molecular chaperone HSP47 in fibroblasts, we examined HSP47 expression using immunofluorescence (Figure 5E) and co‐localized it with α‐SMA. The results demonstrated that L9 reduced the number of fibroblasts expressing HSP47 by 88% (Figure 5F).

### 2.6 L9 Regulates HSP47 Expression Through the IL‐17RA‐Mediated JNK‐HSF1 Pathway

**FIGURE 6:**
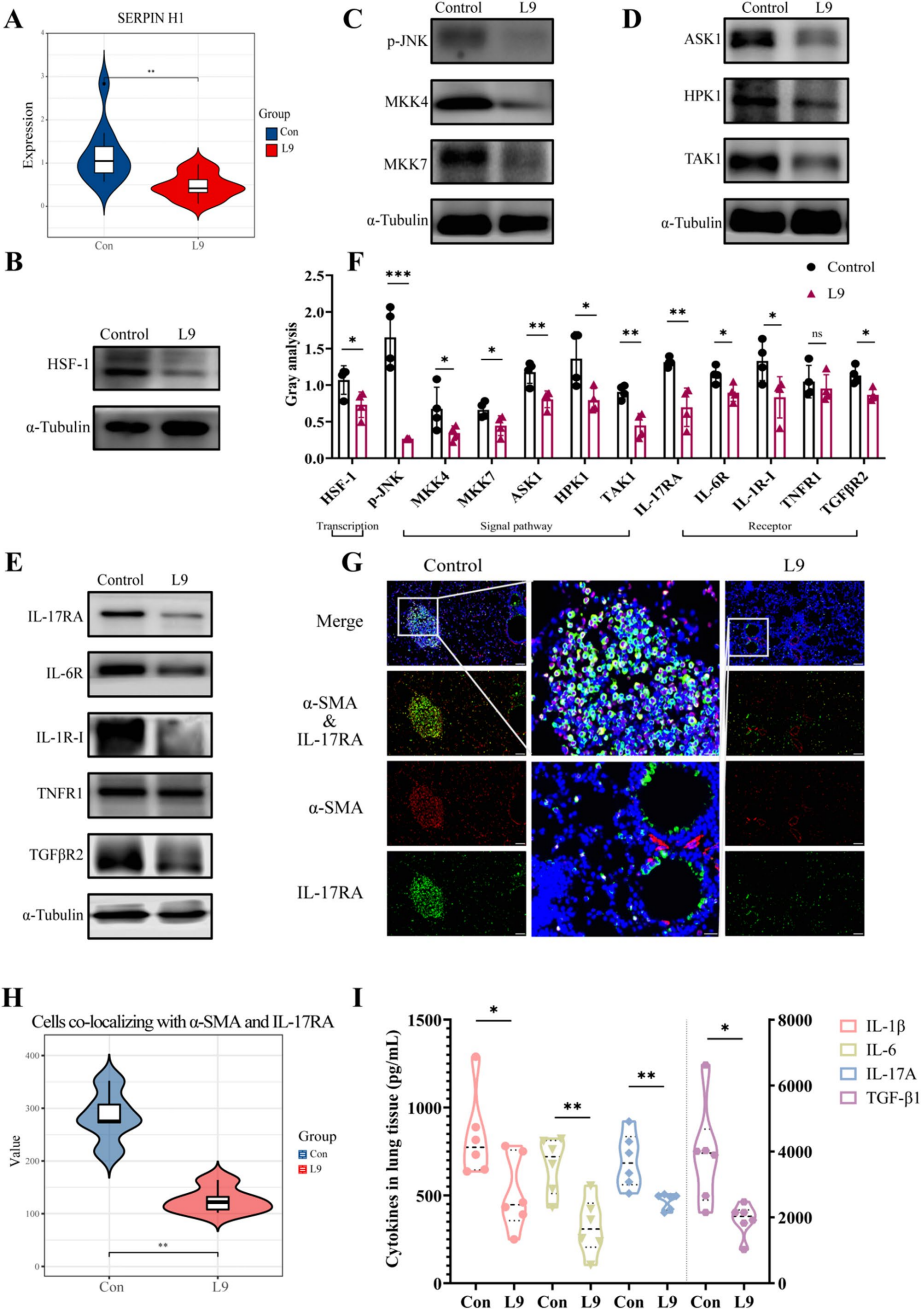
Col‐I production is regulated by L9 via the JNK‐HSF1 pathway. (A) qRT‐PCR analysis of HSP47 expression in mouse lung tissues. Three independent experiments were performed, *n* = 4. (B–E) The expression levels of HSF‐1, p‐JNK, JNK, MKK4, MKK7, ASK1, HPK1, TAK1, IL‐17RA, IL‐6R, IL‐1R‐I, TNFR1, PDGFRβ, TGFβR2, and α‐Tubulin were detected using Western blot analysis. (F) α‐Tubulin was used as the internal reference for HSF‐1, MKK4, MKK7, ASK1, HPK1, TAK1, IL‐17RA, IL‐6R, IL‐1R‐I, TNFR1, PDGFR β and TGFβR2. JNK was used as the internal reference for p‐JNK. Relative protein expression levels were determined using gray scale analysis. Three independent experiments were performed, *n* = 4. (F) The expression levels of IL‐1β, IL‐6, IL‐17A and TGFβ1 in lung tissue, three independent experiments were performed, *n* = 6. (G) Lung sections were stained with immunofluorescence for IL‐17RA and α‐SMA, magnification, 100×. *n* = 4. Statistical analysis was conducted using independent samples *t*‐test. Scale bar = 50 μm. (H) Counting of cells co‐localizing with α‐SMA and IL‐17RA, *n* = 5. Statistical analysis was conducted using independent samples *t*‐test. Scale bar = 50 μm. (I) The expression levels of IL‐1β, IL‐6, IL‐17A and TGFβ1 in lung, three independent experiments were performed, *n* = 6. * *p* < 0.05, ** *p* < 0.01, *** *p* < 0.001.

We ascertained the transcription level and transcription factors of HSP47 to investigate the rationale behind L9 impact on the level of HSP47 protein expression. The *SERPIN H1* gene encodes HSP47. The results (Figure 6A) showed that the L9 group had a significant 61% decrease of *SERPIN H1* gene expression. Consequently, we further investigated the key transcription factor of *SERPIN H1*, HSF1, and discovered that L9 significantly reduced HSF1 levels by 27% (*p* < 0.05) compared to the control group (Figure 6B,F). One of the most prominent HSF1 regulatory pathways is the JNK pathway, which has the capacity to directly phosphorylate the transcription activation domain of HSF1 (Park and Liu 2001). As a result, we detected key proteins on the JNK pathway to identify the proteins in which L9 has a function. Results (Figure 6C,F) showed that L9 strongly suppressed 85% of the phosphorylation activation of the JNK pathway (*p <* 0.05). As is widely known, MKK4 and MKK7 protein regulate JNK to enter the nucleus. The results demonstrate that L9 reduced 43% of MKK4 expression and 22% of MKK7 expression (Figure 6C,F).

ASK1, which regulates MKK4/7 independently of TAK1 and HPK1 proteins, can be further modulated by cytokine receptors (e.g., interleukin [IL]) (Nishitoh et al. 1998). TAK1—mediated regulation of MKK4/7 is induced by the upstream kinase HPK1 (50), which also relays signaling cascades from growth factors (e.g., TGFβ1) (Boomer and Tan 2005). To ascertain the mechanism underlying L9‐mediated regulation of the JNK pathway, our investigation examined the expression of its upstream regulatory proteins. Results demonstrated that L9 intervention significantly reduced (*p <* 0.05) the expression of ASK1, TAK1, and HPK1. Specifically, L9 decreased the expression of TAK1 by 50%, HPK1 by 41%, and ASK1 by 31% (Figure 6D,F).

In particular, cells in the lung that must strictly regulate matrix remodeling and inflammatory responses to prevent abnormal tissue repair are harmed by the senescence‐associated secretory phenotype (SASP), which is made up of aging‐related cytokines, matrix remodeling proteases, and growth factors produced by aging cells (especially epithelial cells and fibroblasts) in aging individuals (Schafer et al. 2017). When TGFβ1 is present, SASP factors IL‐6 and IL‐1β promote the differentiation of naïve CD4^+^ T cells into the TH17 phenotype (Wang et al. 2024). Previous studies have shown that L9 can reduce the level of IL‐17A in the lungs (Wang et al. 2017). Therefore, we detected the SASP‐related receptors upstream of HPK1 and ASK1. Western blot results (Figure 6E,F) demonstrated that the expressions of IL‐17RA, IL‐6R, IL‐1R‐I, and TGFβR2 were significantly inhibited (*p <* 0.05) after L9 intervention, whereas the change in TNFR1 was not significant. Among them, the change in IL‐17RA was the most significant.

### 2.7 L9 Reduced the Number of Th17 Cells in the Lungs and the Level of IL‐17

IL‐17RA is a receptor for inflammatory factors, and such receptors are involved in the regulation of ASK1. Presently, we measured the ligands of the receptors with significant changes (Figure 6I) and found that L9 intervention also significantly decreased the levels of pulmonary IL‐17A, IL‐6, IL‐1β, and TGFβ1 (*p <* 0.05). Among these, the 32% decrease in IL‐17A was the most statistically representative. Therefore, we performed immunofluorescence staining for the IL‐17RA receptor in lung tissues, with co‐localization with α‐SMA, and observed that L9 intervention significantly reduced the number of fibroblasts expressing IL‐17RA by 56% (Figure 6G,H), indicating that IL‐17A is the key factor mediating the effect of L9 on fibroblasts.

### 2.8 L9 Increased the Abundance of SCFA‐Producing Bacteria in the Intestines of Aging Mice

**FIGURE 7:**
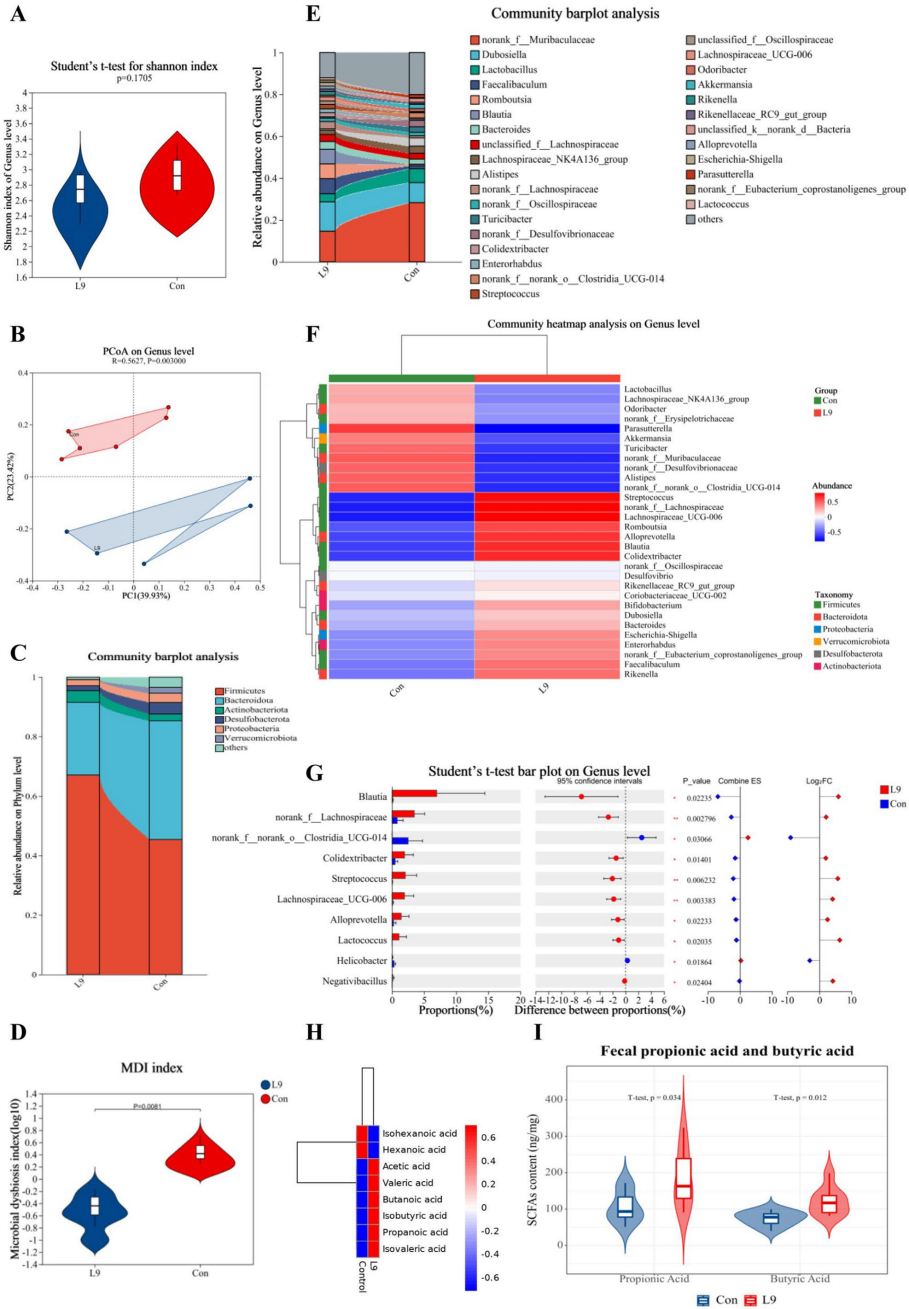
L9 enhanced the number of bacteria that produce SCFA in the intestines of aged mice. (A) Inter‐group difference in the *α* diversity index of mouse gut flora, independent sample *T*‐test, Shannon index. (B) PCoA analysis of intestinal flora in mice at genus level. (C) Phyla level community composition histogram, abundance ranked top 6. (D) Microbial dysbiosis index of mouse feces. (E) Genus horizontal community composition histogram, proportion of abundance top 30. (F) Genera level community composition heat map, abundance ranked top 30, *z*‐score was used for data standardization, species level clustering method average. (G) Genus two‐group difference test, independent sample *t*‐test, 95% confidence interval. (H) Intestinal SCFA grouping content pattern clustering heat map. (I) Intestinal propionic acid and butyric acid content, *n* = 8, independent sample *t*‐test.

Given that L9 primarily impacts the gut microbiota, we first characterized the gut microbiota of mice to explore its role in regulating the gut‐lung axis. Initially, the species abundance and diversity of intestinal microbes in mice were studied, and it was observed that there was little variation in the alpha diversity index between the control group and the L9 group (Figure 7A). The first principal coordinate (PC1) and the second main coordinate (PC2) accounted for 39.93% and 23.42% of the total variation in the intestinal flora community of mice, according to further analysis of the variation overall (Figure 7B). Meanwhile, the examination of the microbial dysbiosis index (MDI) revealed a noteworthy modification in the intestinal flora composition of mice after L9 treatment (*p <* 0.05) (Figure 7D). Thus, additional phylum‐level analysis of the intestinal bacteria that have been identified reveals notable variations between the L9 group and control group, mainly in Firmicutes, Bacteroidota, Actinobacteriota, Desulfobacterota, Proteobacteria, and Verrucomicrobiota (Figure 7C). We next used bar and pie chart analyses to determine the community makeup of the top 30 differentially abundant bacteria at the genus level, and a heatmap to highlight the top 30 differentially abundant genera based on relative abundance (Figure 7E,F). After the L9 intervention, there was a significant drop in potentially harmful bacteria such as norank_f__norank_o__*Clostridia*_UCG‐014, *Acinetobacter*, and *Desulfovibrio*, whereas the abundance of *Dubosiella* and *Lactobacillus* increased (*p <* 0.05). Therefore, by computing the sequencing proportions of various bacterial communities, it (Figure 7G) was observed that the abundance of *Blautia*, norank_f__*Lachnospiraceae*, *Colidextribacter*, *Lachnospiraceae*_UCG‐006, *Rikenella*, and other bacteria showed a substantial upregulation trend in L9 group (*p <* 0.05).

In conclusion, there is a notable increase in the number of genera that produce SCFA following L9 intervention. For instance, by producing SCFA, particularly butyric acid, the species *Blautia* has anti‐inflammatory qualities and affects colonic motility. *Colidextribacter* is positively correlated with the increase of intestinal SCFAs. A positive correlation has been observed between cecal SCFA and *Lachnospiraceae*. Among other short‐chain fatty acids, succinic acid, propionic acid, and acetic acid are typical metabolites of *Rikenella*. To investigate the SCFAs content in the feces, a targeted SCFAs metabolomics examination was carried out. The L9 group's feces were found to include higher concentrations of acetic acid, propionic acid, butyric acid, isobutyric acid, valeric acid, and isovaleric acid (Figure 7H). Propionic acid and butyric acid showed the greatest increases (*p <* 0.05), with propionic acid rising by 74% and butyric acid by 64% (Figure 7I).

### 2.9 L9 Increased the Levels of Propionic Acid and Butyric Acid in the Gut and Serum of Aging Mice

**FIGURE 8:**
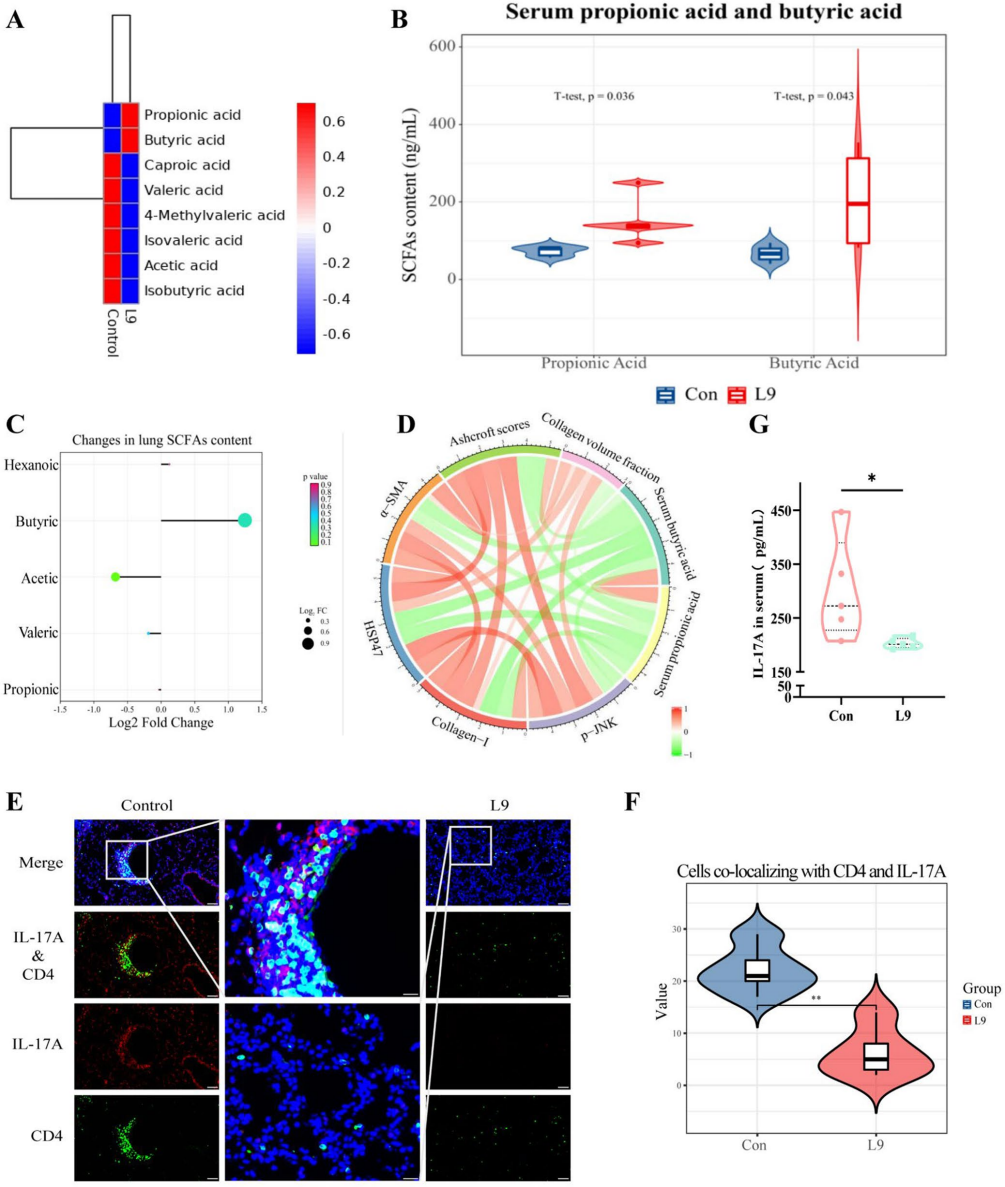
Propionic acid and butyric acid content of the serum and intestines of aged mice were elevated by L9. (A) Serum SCFA grouping content pattern clustering heat map. (B) Serum propionic acid and butyric acid content, *n* = 6, independent sample *t*‐test. (C) Horizontal lollipop of the changes in lung SCFAs content, *n* = 6. (D) Pearson coefficient correlation analysis heat map, two‐tailed, 95% confidence interval. (E) Lung sections were stained with immunofluorescence for IL‐17A and CD4, magnification, 100×. *n* = 4. Statistical analysis was conducted using independent samples *t*‐test. Scale bar = 50 μm. (F) Counting of cells co‐localizing with IL‐17A and CD4, *n* = 5. Statistical analysis was conducted using independent samples *t*‐test. Scale bar = 50 μm. (G) The expression levels of IL‐17A in serum, three independent experiments were performed, *n* = 5. * *p* < 0.05, ** *p* < 0.01.

Ulteriorly, targeted detection of propionic acid and butyric acid levels in serum was performed to determine whether the elevated propionic acid and butyric acid in the intestine enter the systemic circulation. As depicted in Figure 8A,B, serum concentrations of butyric acid and propionic acid in the L9 group were significantly elevated by 193% and 97%, respectively (*p <* 0.05). Accordingly, we hypothesized that propionic acid and butyric acid might exert their effects in the lungs via systemic circulation. However, in the targeted detection of the lungs, we surprisingly found that the concentrations of both propionic acid and butyric acid were below the limit of detection (Figure 8C). Subsequently, correlation analysis revealed a substantial positive correlation (*p <* 0.05) between propionic acid and butyric acid expression (Figure 8D). In addition, there is a favorable link between the improvement in PF and the increase in serum propionic acid and butyric acid levels (*p <* 0.05), with butyric acid exhibiting a stronger correlation (Figure 8D). As key immunomodulatory metabolites, studies have demonstrated that SCFAs may directly affect peripheral immune cells, which are subsequently recruited to the lungs to exert their functions (Espírito Santo et al. 2021). Thus, we hypothesized that circulating propionic acid and butyric acid might modulate the recruitment of immune cells and inflammatory factors to the lungs, thereby mitigating PF. To validate this hypothesis, given that IL‐17A—primarily secreted by Th17 cells—was identified as the key affected factor in our prior findings, we further quantified Th17 cell numbers in lung tissues and observed a 71% reduction following L9 intervention (Figure 8E,F). We subsequently measured circulating IL‐17 levels, which revealed a significant 33% decrease (Figure 8G). Collectively, these data suggest that propionic acid and butyric acid regulate circulating IL‐17A levels, which in turn exerts a downstream effect on the lungs.

### 2.10 Propionic Acid and Butyric Acid Suppress Th17 Cell Differentiation, Thereby Alleviating PF

**FIGURE 9:**
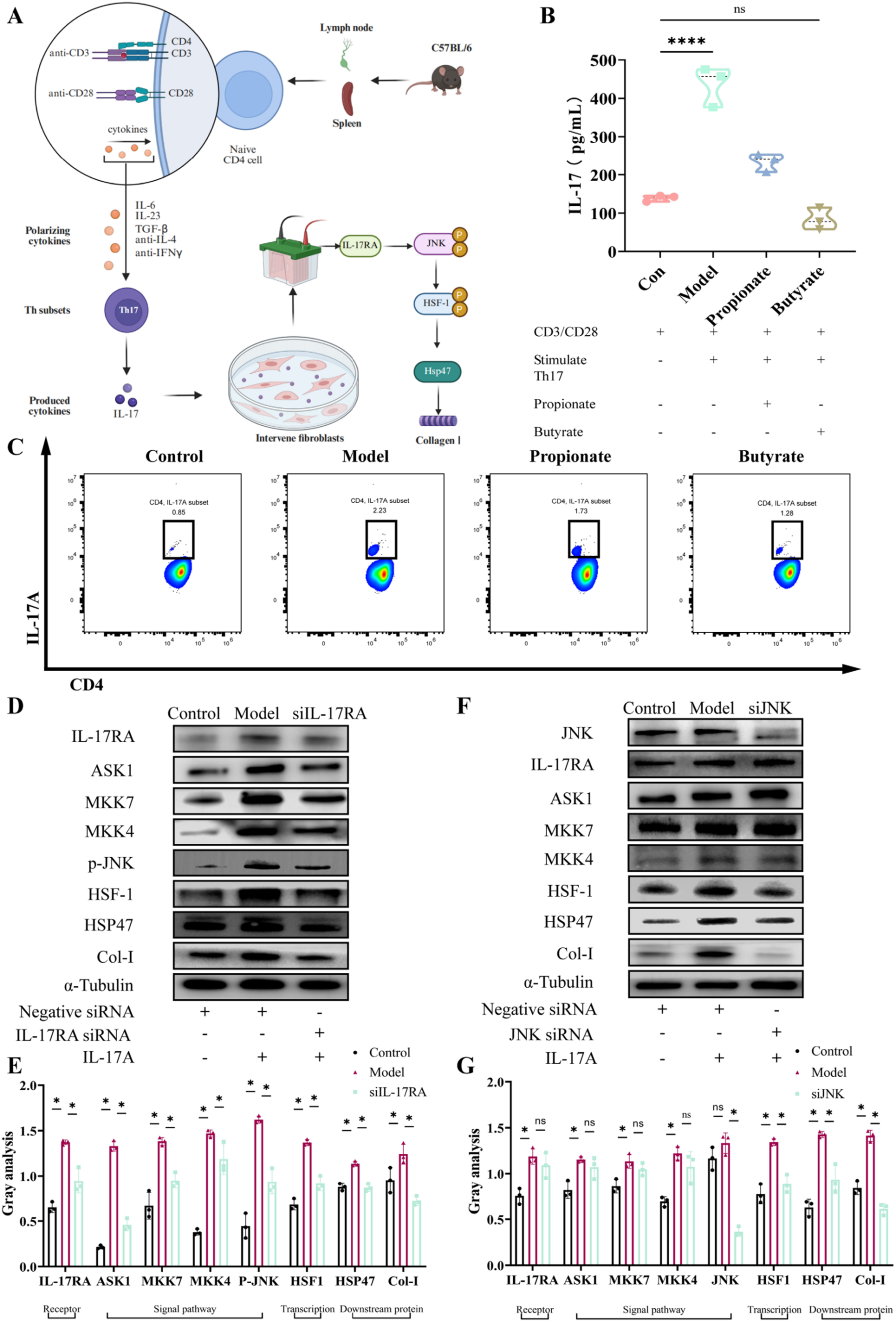
SCFAs inhibit the fibrosis in vitro. (A) Cell Experiment Flow Chart. (B) The expression levels of IL‐17A in culture supernatant, three independent experiments were performed. (C) Representative flow cytometry plots illustrating the in vitro differentiation of naïve CD4+ T cells into Th17 cells under SCFA (propionate and butyrate) treatment. (D, F) The expression levels of IL‐17RA, ASK1, MKK4, MKK7, p‐JNK, HSF1, HSP47, Col‐I and α‐Tubulin were detected using Western blot analysis. (E, G) α‐Tubulin was used as the internal reference for IL‐17RA, ASK1, MKK4, MKK7, p‐JNK, HSF1, HSP47 and Col‐I. Relative protein expression levels were determined using gray scale analysis. * *p* < 0.05, **** *p* < 0.0001.

Based on previous results, we hypothesized that propionic acid and butyric acid may inhibit the differentiation of naïve CD4^+^ T cells into the Th17 phenotype. Naïve CD4^+^ T cells were isolated from mice and stimulated with IL‐6 and IL‐1β in the presence of TGFβ1 to mimic the SASP‐induced microenvironment for Th17 differentiation (Figure 9A). We characterized the effects of propionic acid and butyric acid on Th17 cell differentiation using ELISA and flow cytometry. The results showed that propionic acid and butyric acid reduced IL‐17A production by 46% and 81% (*p <* 0.05), respectively (Figure 9B). Meanwhile, both propionic acid and butyric acid suppressed Th17 differentiation from naïve CD4^+^ T cells, with butyric acid exerting a more potent inhibitory effect (Figure 9C). In summary, propionic acid and butyric acid inhibit Th17 cell differentiation, thereby reducing IL‐17A production.

Previous findings demonstrated that IL‐17A levels were elevated in both the blood and lungs, which was closely associated with T cell differentiation. We further hypothesized that IL‐17A might act as a key factor in activating the JNK‐HSF1 pathway in downstream fibroblasts. However, cell culture supernatants containing IL‐17A failed to exclude the confounding effects of SCFAs, IL‐6, and IL‐1β—all of which function as pro‐differentiation factors (Figure 9A). Thus, we directly supplemented IL‐17A into the culture medium of NIH/3T3 cells to mimic the pulmonary microenvironment, aiming to clarify its impact on Col‐I production. Western blot analysis (Figure 9D,F) revealed that IL‐17A supplementation increased Col‐I production (*p <* 0.05). To definitively establish the causal relationship of the IL‐17RA‐mediated signaling cascade, we performed loss‐of‐function experiments using small interfering RNA (siRNA) transfection in NIH/3T3 fibroblasts. First, fibroblasts were transfected with IL‐17RA‐specific siRNA prior to IL‐17A stimulation. As shown in Figure 9D,E, silencing IL‐17RA significantly abrogated the IL‐17A‐induced upregulation of the receptor itself, as well as the entire downstream cascade, including ASK1, MKK7, MKK4, p‐JNK, HSF1, HSP47, and ultimately Col‐I synthesis (*p* < 0.05). Next, to confirm the specific role of JNK as the central intracellular mediator, cells were co‐transfected with siRNAs targeting both JNK1 and JNK2. Following IL‐17A stimulation, the expression levels of the upstream components (IL‐17RA, ASK1, MKK7, and MKK4) remained highly elevated, showing no significant difference compared to the model group (Figure 9F,G). However, JNK was profoundly suppressed, effectively severing the signal transduction. Consequently, the downstream activation of HSF1, the induction of the molecular chaperone HSP47, and the synthesis of Col‐I were significantly reversed to basal levels (*p* < 0.05). These genetic intervention data mechanistically prove the unidirectional causal flow of the IL‐17RA–JNK–HSF1–HSP47 axis in driving collagen production. Meanwhile, this confirms that IL‐17A fundamentally and directly alters the intracellular activation state of individual fibroblasts, independent of cell proliferation.

## 3 Discussion

Previous studies found that the incidence of PF significantly increases with age (Quan et al. 2024). Initially, we confirmed that with aging, there is an increased deposition of pulmonary ECM in aging mice and older populations, resulting in a PF phenotype. Recent studies indicate that Lactobacillus is closely associated with collagen synthesis (Yu et al. 2024). L9, a probiotic isolated from the intestines of healthy elderly individuals with longevity, belongs to the genus Lactobacillus (Wang et al. 2017). Further analysis confirmed that PF in aging mice was indeed reversed through oral intake of L9. Mechanistically, within the gut‐lung axis, L9 reshapes the gut microbiota by enhancing the abundance of SCFA‐producing bacteria, leading to increased production of propionic and butyric acids that enter the bloodstream. These SCFAs regulate Th17 cell differentiation and their migration to the lungs, thereby reducing IL‐17A production. The decreased IL‐17A subsequently downregulates IL‐17RA receptor expression on pulmonary fibroblasts, suppressing activation of the JNK‐HSF1 signaling pathway. This cascade results in reduced Col‐I synthesis, decreased ECM deposition in the lung stroma, and ultimately alleviation of PF.

Col‐I and Col‐III comprise approximately 90% of total lung collagen and play a dominant role in the pathogenesis of PF (Calabresi et al. 2007). Our results indicate that with advancing age, both the gene and protein expression levels of Col‐I and Col‐III significantly increase. Interestingly, while changes in Col‐I are widely recognized in studies of PF progression, the changes in Col‐III have shown inconsistent results (Henderson et al. 2020; Quan et al. 2024; Calabresi et al. 2007; Tieyuan et al. 2022). Recent evidence indicates that although Col‐III supports early matrix formation and basal structural integrity, pathological stiffness in age‐related PF is primarily driven by excessive Col‐I cross‐linking (Quan et al. 2024). While both Col‐I and Col‐III are often upregulated in PF (Tieyuan et al. 2022), their roles are distinct; reduced Col‐III can even exacerbate fibrosis by promoting α‐SMA expression and scar formation (Volk et al. 2011). Therefore, the lack of Col‐III modulation by L9 highlights a high degree of therapeutic precision: by downregulating the Col‐I‐biased chaperone HSP47, L9 specifically targets pathological rigidity without indiscriminately degrading the Col‐III networks required for basic tissue homeostasis. Notably, L9 acts primarily by inhibiting Col‐I synthesis rather than promoting its degradation. Given that L9 alleviates PF in mice by suppressing the expression of HSP47, a protein critical for procollagen triple helix folding during collagen biosynthesis (Roque 2022), this result also validates the hypothesis of Sakamoto et al. (2023) that HSP47 is a promising therapeutic target for PF.

Recent investigations have validated the efficacy of JNK inhibitors in preclinical PF models (Diwan et al. 2024). JNK pathway also serves as the primary regulator of HSF‐1, the transcription factor of HSP47 (Morano and Thiele 2018). Mechanistically, we confirmed that L9 attenuates Col‐I biosynthesis by disrupting the JNK‐HSF1 signaling axis. It is noteworthy that age‐related PF is associated with chronic inflammatory stress (Moss et al. 2022), which differs from acute cellular responses that mainly depend on transient kinase phosphorylation. Sustained exposure to IL‐17A induces profound transcriptional and post‐transcriptional remodeling in target cells (McGeachy et al. 2019). Specifically, chronic IL‐17A signaling creates a positive feed‐forward loop that upregulates the transcription of its own receptor (IL‐17RA) to continuously sensitize fibroblasts (McGeachy et al. 2019). Concurrently, the IL‐17 receptor complex recruits RNA‐binding proteins, such as HuR, to stabilize the mRNA transcripts of downstream mediators—including MAP3Ks like ASK1—effectively preventing their degradation (Herjan et al. 2018). Therefore, the increased total protein levels of ASK1 (Valenca et al. 2022) and IL‐17RA observed in fibrotic lungs may reflect an adaptive response to chronic stress that helps maintain pro‐fibrotic signaling. Consequently, the reduction of these proteins after L9 treatment suggests that this sustained transcriptional and post‐transcriptional drive is attenuated.

sRecent studies have focused on the gut‐lung axis to explain how orally administered substances influence the lungs. Deng et al. (2021) demonstrated that oral administration of L9 increased the abundance of butyrate‐producing bacteria in mice, thereby reducing pulmonary inflammation. This effect was recapitulated by oral sodium butyrate treatment. Lu et al. (2018) reported that oral L9 supplementation in piglets elevated fecal acetic, propionic, butyric, and valeric acids. Our research further characterized that oral L9 administration increased circulating propionate and butyrate content in mice. Previous studies have established that these SCFAs entering the bloodstream directly impact metabolism and function in organs/tissues such as the lung, liver, and skeletal muscle (Boets et al. 2017). However, the concentrations of SCFAs in the lungs were below the limit of detection. We recognize that standard gas chromatography–mass spectrometry (GC–MS) may lack the requisite sensitivity to quantify trace, highly volatile SCFA concentrations within a highly aerated and metabolically active tissue like the lung. Furthermore, this is potentially attributed to the metabolic characteristics of SCFAs as cellular energy sources for colonic cells (Vieira et al. 2019). Most existing studies focus on the immune‐related pulmonary effects indirectly mediated by SCFAs. For instance, propionate and butyrate inhibited Th9 cell differentiation and reduced their migration and infiltration into the lungs by upregulating FOXP3 expression in circulating CD4^+^ T cells, thereby alleviating OVA‐induced pulmonary inflammation in mice (Vieira et al. 2019). Atarashi et al. (2013) revealed that modulating specific intestinal bacteria to produce SCFAs effectively induces CD4^+^FOXP3^+^ regulatory T cell differentiation and upregulates anti‐inflammatory molecules, including IL‐10 and ICOS. Chakraborty et al. (2017) demonstrated that butyrate reverses cardiolipin‐induced inhibition of IL‐10 expression in pulmonary myeloid‐derived suppressor cells by inhibiting HDAC activity, reduces the pro‐inflammatory cytokine TNF in the lungs, and alleviates inflammatory injury in a bacterial pneumonia model (Chakraborty et al. 2017). Taken together, our findings also indicate that the immunomodulatory effects of SCFAs are systemic rather than resulting from local accumulation in the lungs. Therefore, the observed reduction in lung Th17 cells is more likely attributable to a systemic effect, whereby SCFAs suppress Th17 differentiation in peripheral lymphoid tissues, leading to a reduced circulating pool of Th17 cells. This systemic reduction would, in turn, be expected to limit the recruitment and migration of Th17 cells into the lung, thereby decreasing local IL‐17A levels and downstream fibrotic responses. While SCFAs represent a major, highly quantifiable pathway driving Th17 suppression, we cannot entirely exclude the concurrent synergistic contributions of other microbiome‐derived factors. For instance, circulating bacterial extracellular vesicles are increasingly recognized as vital mediators of distant immune modulation along the gut‐lung axis (Özçam and Lynch 2024). Additionally, shifts in secondary bile acid metabolism have been proven to directly control Th17 and Treg cell differentiation (Hang et al. 2019). These alternative pathways warrant thorough exploration in future studies.

Intriguingly, evidence indicates that SCFAs modulate the differentiation of circulating immunocytes (Trompette et al. 2014), which subsequently migrate to lung tissue and polarize into selectively activated phenotypes (Landsman and Jung 2007). JNK pathway is mainly stimulated by cytokines and chemokines (Bogoyevitch et al. 2010), many of which are components of the SASP implicated in age‐related PF (Wang et al. 2024). Previous studies have reported that oral administration of L9 reduced the IL‐17A‐driven pro‐inflammatory immune response in the lungs (Wang et al. 2017). Consistently, IL‐17A has been well‐documented to mediate inflammation, fibrosis, and cellular senescence (Wang et al. 2024). Our results revealed that L9 intervention significantly reduced pulmonary levels of IL‐1β, IL‐6, IL‐17A, and TGF‐β1, with the most pronounced effect on IL‐17A. Th17 cells, the primary producers of IL‐17A/F (Nie et al. 2022), and their accumulation have been reported in the lungs of PF patients (Celada et al. 2018). Consistently, we observed a marked decrease in pulmonary Th17 cells following L9 treatment. Notably, increased abundance of SCFA‐producing gut microbiota has been inversely correlated with IL‐17A levels (Pagliai et al. 2011). A recent skin fibrosis study demonstrated that SCFAs exerted therapeutic effects comparable to anti‐IL‐17 monoclonal antibodies (Chen et al. 2024). In vitro, propionate and butyrate (particularly) inhibited naïve CD4^+^ T cell differentiation into Th17 cells, thus reducing IL‐17 levels. In summary, this study uncovers a novel gut‐lung axis‐mediated anti‐fibrotic pathway, wherein the probiotic L9 reshapes the gut microbiota to augment the production of propionic and butyric acids. These SCFAs inhibit Th17 cell differentiation, thereby suppressing the IL‐17RA‐mediated JNK‐HSF1 signaling cascade in fibroblasts, ultimately reducing Col‐I deposition and alleviating age‐related PF. These findings highlight the potential of L9 as a microbiome‐targeted precision nutrition strategy for probiotic‐based intervention, providing novel insights to advance research in the challenging field of age‐related PF. These findings generate a robust hypothesis that necessitates future prospective human cohorts involving matched fecal metabolomics and bronchoalveolar lavage profiling to definitively validate the gut‐lung SCFA‐Th17 axis in elderly populations. Future investigations should subsequently evaluate the safety and efficacy of L9 as an adjuvant to existing therapeutics in PF patients stratified by gut microbiota profiles—specifically those with low SCFA‐producing communities or dysbiotic Th17‐promoting flora. Additionally, studies are warranted to assess how such precision nutrition approaches optimize therapeutic efficacy and mitigate interindividual variability. Such endeavors will facilitate the translation of microbiota‐targeted strategies into personalized care for age‐related PF.

## 4 Materials and Methods

### 4.1 Study Design

This study aimed to investigate how L9 mitigates age‐related PF. Verify the fibrotic lesions that occur in the lungs during the aging process through human lung tissue samples. A total of 8 lung cancer patients participated in this study. The patients were divided into a young group (≤ 40 years) and an elderly group (≥ 60 years), following the method outlined by Quan and colleagues (Quan et al. 2024). Human paratumor tissues were collected in accordance with the Code of Ethics of the World Medical Association (declaration of Helsinki), and the protocol was approved by the Human Research Ethics Committee of China Agricultural University (approval number CAUHR‐20230502). Informed consent has been obtained from the subject after explaining the nature of the study and its possible consequences. Publicly available human and mouse lung bulk RNA‐seq datasets (GEO Accession Nos. GSE1642 and GSE123293) were downloaded for bioinformatics analysis. Natural aging C57BL/6 mice (15–24 months old) were used to evaluate the effects of L9 on age‐related PF. Mice were randomly divided into the control group and L9 treatment group. Equal numbers of male and female mice were included to reduce gender‐associated experimental bias. Each mouse was considered an independent experimental unit (N). All animals were housed under specific pathogen‐free conditions with ad libitum access to food and water. The experimental protocol was approved by the Institutional Animal Care and Use Committee of China Agricultural University. No sex‐based subgroup analysis was conducted in this study. Our primary objective was to authentically replicate the physiological state of natural senescence. For PF phenotyping, histological and biochemical analyses (e.g., Masson's trichrome staining, Western blotting for Col‐I) were performed to assess PF severity. Through the study of the biosynthesis flux of Col‐I protein, we have elucidated the molecular mechanism underlying the alleviation of PF following L9 intervention. 16S rRNA gene sequencing was used to profile gut microbial composition. Fecal and serum SCFAs were quantified using gas chromatography–mass spectrometry (GC–MS). Naïve CD4^+^ T cells were isolated and differentiated into Th17 cells with/without SCFA treatment. IL‐17A‐stimulated lung fibroblasts were used to validate JNK pathway activation and subsequent collagen synthesis.

### 4.2 Animals

Beijing Vital River Laboratory Animal Technology Co. Ltd. supplied 16 male and female C57BL/6 J mice, aged 15 months, that were specific‐pathogen‐free (SPF) and weighed 35–40 g. The mice were kept in a room with 22°C ± 2°C temperature and 55% ± 5% humidity. Simultaneously, the 12‐h cycle of day and night is rigidly enforced by the mouse housing. The mice were fed in an adapted manner for 1 week prior to the official experiment. Every mouse received a regular diet and had unrestricted access to food and water. The Animal Welfare and Ethics Committee of China Agricultural University granted approval for the animal experiment (Approval No.: AW32504202‐5‐8). Two groups of eight mice each—the L9 group and the control group—were randomly selected from among the mice. At 15 M, 4 mice were euthanized. From the start of the formal experiment on 15 M mice, 100 μL of L9 suspension (4 × 10^9^ CFU/mL) was delivered gastric to each L9 group at 3 p.m. daily, while the control group received the same volume of normal saline intraperitoneally. Following 9 months of treatments, the mice were euthanized.

### 4.3 Histology

The top lobe of the mouse lung was fixed in 4% paraformaldehyde (BL539A, Biosharp, Beijing, China) solution for 48 h and then washed with tap water for 4 h to remove the paraformaldehyde. The lung tissue was then sectioned, progressively dehydrated, and immersed in ethanol (213‐3, LookChem, Tianjin, China) at varying concentrations from low to high, followed by xylene (S1763, Keao, Beijing, China) until translucent. The tissue was then placed in a combination of xylene and paraffin and heated to 60°C for 2 h to melt the paraffin. After cooling, it was sectioned with a tissue slicer (Leica CM30505, Leica, Nussloch, Germany).

The kit was used for HE staining (G1120, Solarbio, Beijing, China), Masson staining (G1340, Solarbio, Beijing, China), and Sirius red staining (G1472, Solarbio, Beijing, China) in accordance with the manufacturer's instructions. An optical microscope (XSP‐8CA, Yoke Instrument, Shanghai, China) was used to examine the tissue slices. The Ashcroft pulmonary fibrosis scores (Ashcroft et al. 1988) were determined to evaluate fibrosis in a blinded manner. Using a polarized‐light microscope (DM4P, Leica, Germany), the sections dyed with Sirius red were examined.

### 4.4 Cell Culture

Naïve CD4^+^ T cells were isolated from spleens and pooled lymph nodes (inguinal, axillary, and superficial cervical) of 8‐week‐old C57BL/6 mice using the EasySep Mouse Naïve CD4^+^ T Cell Isolation Kit (19765, Stemcell) according to the manufacturer's instructions. Cells were resuspended in RPMI‐1640 medium (abs9484, Absin) supplemented with 2 mM L‐glutamine, 2 g/L sodium bicarbonate, 2 g/L D‐glucose, 5 mg/L phenol red, and 10% FBS (KGL3006‐10, Keygen), then activated with Dynabeads Mouse T‐Activator (11456, Thermo). Th17 differentiation was induced by culturing cells for 4 days in the presence of recombinant mouse IL‐6 (50 ng/mL), TGF‐β1 (2 ng/mL), IL‐23 (5 ng/mL), anti‐mouse IL‐4 (10 μg/mL), and anti‐mouse IFN‐γ (10 μg/mL). Cells were subsequently stimulated with Cell Stimulation Cocktail (004970, Thermo) containing PMA and ionomycin for 5 h. Culture supernatants were collected by centrifugation at 300 *g* for 10 min and stored at −20°C for ELISA analysis.

NIH/3 T3 (CRL‐1658, ATCC) cells were purchased from the Wuhan Procell Life Science Technology Co. Ltd. NIH/3T3 cells were cultured in DMEM + 10% NCS + 1% P/S (CM0171, Procell).

### 4.5 siRNA Transfection

To validate the signal transduction pathway, NIH/3T3 fibroblasts were seeded into 6‐well plates and cultured until reaching approximately 60%–70% confluence. Small interfering RNAs (siRNAs) specifically targeting mouse IL‐17RA, JNK1, and JNK2, along with a negative control (NC) siRNA, were utilized for transient transfection. For the IL‐17RA knockdown group, cells were transfected with 30 pmol of IL‐17RA siRNA per well. For the JNK knockdown group, to concurrently silence both isoforms and prevent compensatory effects, cells were co‐transfected with a mixture of JNK1 and JNK2 siRNAs at a combined final quantity of 30 pmol per well in the 6‐well plate. Transfections were performed using Lipofectamine RNAiMAX (13778100, Thermo) according to the manufacturer's protocol. Following 8 h of transfection, the culture medium was replaced, and the fibroblasts were stimulated with recombinant mouse IL‐17A (10 ng/mL) for further cultivation. The knockdown efficiency and the expression of downstream signaling proteins were subsequently evaluated by Western blot analysis.

### 4.6 Western Blot

For the purpose of analyzing lung tissue samples, 80–100 mg of fresh tissue were combined with 1 mL of lysis buffer (R0010, Solarbio, Beijing, China), 10 μL of PMSF (P0100, Solarbio, Beijing, China), and 10 μL of protein phosphatase inhibitor (P1260, Solarbio, Beijing, China) and incubated on ice for 30 min. The tissue was then homogenized and centrifuged. Each sample's high‐molecular‐weight proteins were separated using SDS‐PAGE. A 4°C overnight incubation of the primary antibody was performed for α‐Tubulin (ab7291, Abcam) p21 (28248‐1‐AP, Proteintech), p16 (28416‐1‐AP, Proteintech), Collagen I (ab260043, Abcam), Collagen III (ab184993, Abcam), α‐SMA (ab184705, Abcam), LOXL2 (ab96233, Abcam), LOX (ab174316, Abcam), Cathepsin K (ab187647, Abcam), MMP‐1 (ab137332, Abcam), MMP‐2 (ab92536, Abcam), P5CS (17719‐1‐AP, Proteintech), PSAT‐1 (10501‐1‐AP, Proteintech), PHGDH (14719‐1‐AP, Proteintech), Col1α1 (#72026, CST), Col1α2 (ab308221, Abcam), Cleaved PICP (ab260043, Abcam), HSP47 (10875‐1‐AP, Proteintech), HSF‐1 (51034‐1‐AP, Proteintech), p‐JNK (4668T, CST), JNK (9252T, CST), MKK4 (17340‐1‐AP, Proteintech), MKK7 (55030‐1‐AP, Proteintech), ASK1 (ab45178, Abcam), HPK1 (4472S, CST), TAK1 (12330‐2‐AP, Proteintech), PDGFRβ (13449‐1‐AP, Proteintech), and TGFβR2 (ab259360, Abcam), at a 1:1000 dilution. This was then followed by a 1‐h incubation with the corresponding HRP conjugated secondary antibodies at a 1:10,000 dilution. A chemiluminescence imaging equipment (ChemiScope 6100, Clinx Science Instruments, Shanghai, China) was used to identify and view the bound antibodies. There were three duplicates in every group.

### 4.7 Immunofluorescence Staining

Following deparaffinization and hydration, tissue slices embedded in paraffin were submerged in sodium citrate antigen retrieval solution (C1031, Solarbio, Beijing, China) and microwave‐boiling. Following a 15‐min immersion in a PBS solution containing 1% Triton X‐100 (T8200, Solarbio, Beijing, China), the sections were blocked for 1 h at room temperature using 10% goat serum (C01‐03001, Bioss, Beijing, China). After that, primary antibodies were added and incubated overnight at 4°C. These included LOXL2 (ab96233, Abcam), LOX (ab174316, Abcam), HSP47 (10875‐1‐AP, Proteintech), and PDGFRβ (13449‐1‐AP, Proteintech). The slices were treated with secondary antibodies the next day for 1 h at room temperature and in the dark. The sections were then mounted using an anti‐fluorescence quenching sealing solution (P0126, Beyotime, Shanghai, Beijing) after being stained for 10 min with a DAPI working solution (C0065, Solarbio, Beijing, China). A laser confocal microscope (LSM900, Zeiss, Nussloch, Germany) was used for observation and photography.

### 4.8 Enzyme‐Linked Immunosorbent Assay

We followed the directions on the kit exactly when doing the enzyme‐linked immunosorbent test (ELISA). To homogenize the lung tissues (0.1 mg), a tissue homogenizer (FastPrep‐24, MP Biomedicals, Solon, OH, USA) was used in 1 mL of PBS. After that, the material underwent PINP analysis (E‐EL‐M0233, Elabscience), IL‐6 (EK206, Multisciences) and IL‐1β (EK201B, Multisciences). ELISA assays were performed on IL‐17A (EK217, Multisciences) and TGFβ1 (EK981, Multisciences) in mouse lung tissue and serum samples. The supernatant of mouse cell culture was detected using the Mouse IL‐17 ELISA Kit (EK217, Multisciences).

### 4.9 RNA Extraction and RT‐qPCR

Using pre‐chilled Trizol reagent (15596018CN, Thermo), lung tissue samples and cells were lysed for 30 min as part of the RNA extraction procedure. RNA was then isolated in accordance with the manufacturer's instructions. A NanoDrop spectrophotometer (Thermo Scientific, Madison, WI, USA) was used to measure the amount of RNA. The cDNA Reverse Transcription Kit (G592, abm, Zhenjiang, China) was then used to synthesize cDNA. Using SYBR Green (A25742, Thermo) as the fluorescent dye, an RT‐qPCR was carried out for a gene expression study using a real‐time PCR machine (QuantStudioTM 5, Thermo Scientific, Marsiling Industrial Estate, Singapore). Six biological replicates and three technical replicates made up each group. Using α‐tubulin as the reference gene for normalization, the fold change values were computed using the 2^∆∆*Ct*^ method and displayed as fold changes. The primers used for gene assessment were as follows:

*α‐TUBULIN* F CCTAAACAGGTTGATAGGCCAAA.

*α‐TUBULIN* R CTCGCCTTCCACAGAATCCA.

*SERPIN H1* F CAACCCCTTTGACCAAGACA.

*SERPIN H1* R TGATTATCTCGCACCAGGAA.

### 4.10 16S rRNA Sequencing

16S rRNA sequencing was conducted using liquid nitrogen extracted from mouse feces. Based on the previously described methodology, 16S rRNA genes in bacterial communities within intestinal contents were altered. Using 338F and 806R primers, the 16S rRNA gene (V3‐V4 region) was the target for DNA preparation on the Illumina MiSeq sequencing platform.

Forward primer: 5′‐ACTCCTACGGGAGGCAGCAG‐3′ (338F).

Reverse primer: 5′‐GGACTACHVGGGTWTCTAAT‐3′ (806R).

All the OTU sequence was divided using Uparse (version 7.0.1090, http://drive5.com/uparse/). Database employing Qiime platform (http://qiime.org/scripts/assign_taxonomy.html), Silva Release 138 (16S bacterium) (http://www.arb‐silva.de). RDP Classifier (Version 2.11, http://sourceforge.net/projects/rdp‐classifier/) uses a bayesian method and has a confidence threshold of 0.7, making it comparable to the 97% level.

### 4.11 SCFA Targeting Assay

To estimate fecal SCFA, a little modification was made to the reference technique. For testing, 20 mg of fecal samples were taken from each sample. 2‐Ethyl butyric acid served as the internal standard, while n‐butanol was utilized to prepare the SCFA mixed standard stock solution and internal standard stock solution. The instrument used was GC/MSD (8890B‐5977B, Agilent Technologies Inc. CA, UAS). Chromatographic settings included a high purity helium carrier gas, a 1.0 mL/min flow rate, and an inlet temperature of 180°C. One microliter of solvent was injected, with a split ratio of 10:1, and the solvent was left to extend for 2.5 min. The column oven's starting temperature was 80°C. It was then set to 120°C at a rate of 20°C per minute, increased to 160°C at a rate of 5°C per minute, and finally ran at 220°C for 3 min. Conditions for mass spectrometry: electron energy of 70 eV, quadrupole temperature of 150°C, transmission line temperature of 230°C, electron bombardment ion source, ion source temperature of 230°C. The ion scanning mode was chosen as the scanning mode. The target SCFA ion fragments were automatically identified and integrated using the Masshunter quantitative software (version number: v10.0.707.0, Agilent Technologies Inc. CA, UAS) default parameters. The standard curve was used to determine each sample's detection concentration as well as the true amount of short‐chain fatty acids present in the sample.

To detect serum SCFA, 50 μL serum samples were collected. Using acetonitrile, a mixed standard stock solution was made. Qualitative and quantitative detection of the target in the sample was accomplished using a liquid mass spectrometer (QTRAP 6500 Plus, AB Sciex, Shanghai, China). Conditions for chromatography: a column temperature of 40°C and an injection volume of 2 μL. Mobile phases A (0.1%) and B (0.1%) of the system are formic acid‐water solution and acetonitrile, respectively. Mass spectrometry conditions: utilizing negative mode detection, Curtain Gas is 35, Collision Gas is Medium, IonSpray Voltage is −4500, Temperature is 450, Ion Source Gas1 is 40, Ion Source Gas2 is 40. SCFA ion fragments are processed by Sciex OS (version number: 3.0, AB Sciex, Shanghai, China) software by default. The concentration that was calculated is as previously stated.

### 4.12 Flow Cytometry

Previously differentiated Th17 cells were stimulated with Cell Activation Cocktail (containing brefeldin A; 423303, BioLegend) for 5 h. After stimulation, the cells were centrifuged at 300 *g* for 5 min to harvest single‐cell suspensions. The single‐cell suspensions were stained with APC anti‐CD4 antibody (100515, BioLegend) at 4°C for 30 min, with the entire process protected from light. Subsequently, the cells were fixed for 40 min using the eBioscience Intracellular Fixation & Permeabilization Buffer Set (Catalog No. 88‐8824‐00, Thermo) following the manufacturer's instructions. After permeabilization, the cells were stained with FITC anti‐IL‐17A antibody (506907, BioLegend) at room temperature for 30 min, again protected from light. Flow cytometric analysis was performed on a spectral flow cytometer (Cytek NL‐CLC3000), and the acquired data were analyzed using FlowJO software (Version 10.10.0).

### 4.13 Statistical Analysis

The mean ± standard deviation (SD) is the reporting metric for all data. The program GraphPad Prism 9 (version 9.0.0(121)) was utilized to create figures and analyze data. The independent sample *t*‐test, which is a two‐tailed test, was used to compare quantitative data between the two groups. Three or more groups were compared using one‐way ANOVA with Tukey's post hoc correction. A significant difference was defined as *p* < 0.05. Statistical significance is indicated by asterisks (*p < 0.05, ** *p* < 0.01, *** *p* < 0.001, and **** *p* < 0.0001). Bar plots with error bars were used to present mean values and variability, facilitating direct comparison of group differences. Violin plots integrated with box‐and‐whisker plots were employed to illustrate data distribution, median, quartiles, dispersion, and outliers. The choice of visualization format was based on data characteristics and the key information to be conveyed. Research design figure was created with BioRender.com.

## Author Contributions

**Ran Bi:** conceptualization, methodology, formal analysis, investigation, visualization, resources, data curation, writing – original draft, writing – review and editing. **Yiran Zhang:** conceptualization, validation, investigation, data curation, visualization, writing – original draft, writing – review and editing. **Wen Zhang:** methodology, Formal analysis, Investigation, Resources, Data Curation, Visualization. **Chenhong Shi:** software, validation, formal analysis, resources, data curation. **Ziyu Qiao:** formal analysis, investigation, visualization, resources, data curation. **Rui Quan:** conceptualization, methodology, software, visualization. **Yanan Sun:** validation, supervision, visualization, project administration. **Juan Chen:** conceptualization, validation, visualization. **Ran Wang:** resources, supervision, project administration. **Fazheng Ren:** supervision, funding acquisition, writing – review and editing. **Yixuan Li:** conceptualization, validation, supervision, project administration, funding acquisition, writing – review and editing. All authors have read and approved the final manuscript.

## Funding

The work was supported by the Young Elite Scientist Sponsorship Program by CAST (2022QNRC001) and the 111 Project of the Education Ministry of China (B18053).

## Conflicts of Interest

The authors declare no conflicts of interest.

## Data Availability

The data that support the findings of this study are available from the corresponding author, Yixuan Li, upon reasonable request. The accession number for the 16S rDNA sequencing data reported in this study is SRA: PRJNA1222143.
